# Mesoscopic Structure and Social Aspects of Human Mobility

**DOI:** 10.1371/journal.pone.0037676

**Published:** 2012-05-31

**Authors:** James P. Bagrow, Yu-Ru Lin

**Affiliations:** 1 Engineering Sciences and Applied Mathematics, Northwestern University, Evanston, Illinois, United States of America; 2 Center for Complex Network Research, Northeastern University, Boston, Massachusetts, United States of America; 3 College of Computer and Information Science, Northeastern University, Boston, Massachusetts, United States of America; 4 Institute for Quantitative Social Science, Harvard University, Cambridge, Massachusetts, United States of America; Universitat Rovira i Virgili, Spain

## Abstract

The individual movements of large numbers of people are important in many contexts, from urban planning to disease spreading. Datasets that capture human mobility are now available and many interesting features have been discovered, including the ultra-slow spatial growth of individual mobility. However, the detailed substructures and spatiotemporal flows of mobility – the sets and sequences of visited locations – have not been well studied. We show that individual mobility is dominated by small groups of frequently visited, dynamically close locations, forming primary “habitats” capturing typical daily activity, along with subsidiary habitats representing additional travel. These habitats do not correspond to typical contexts such as home or work. The temporal evolution of mobility within habitats, which constitutes most motion, is universal across habitats and exhibits scaling patterns both distinct from all previous observations and unpredicted by current models. The delay to enter subsidiary habitats is a primary factor in the spatiotemporal growth of human travel. Interestingly, habitats correlate with non-mobility dynamics such as communication activity, implying that habitats may influence processes such as information spreading and revealing new connections between human mobility and social networks.

## Introduction

Understanding human movement is essential for a range of society-wide technological problems and policy issues, from urban planning [1] and traffic forecasting [2], to the modeling and simulation of epidemics [3], [4], [5]. Recent studies on mobility patterns have shown that spatiotemporal traces are highly non-random [6], [7], [8], exhibiting distinct dynamics subject to geographic constraints [9], [10], [11], [12], [13], [14]. Analytical models have been developed to reflect individual mobility dynamics such as the tendency to move back and forth between fixed locations on a regular basis [15]. When examining populations, movement patterns may be highly correlated with dynamics such as contact preference [9], [11], yet this has not been well studied at the individual level. Previous work on human mobility has focused primarily on simple measures that forego the majority of the detailed information available in existing data. There is good reason for this, as basic approaches tend to be most fruitful for new problems. Yet these measures reduce an entire mobility pattern to a single scalar quantity, potentially missing important details and throwing away crucial information.

A number of approaches are available for studying the geographic substructure of individual mobility. One route is to perform spatial clustering [16] on the specific locations an individual visits, potentially revealing important, related groups of locations. However, such analysis is purely spatial, neglecting the detailed spatiotemporal trajectories available for each person, reducing their mobility to a collection of geographic points and ignoring any information regarding the *flows*, or frequencies of movement, between particular locations. At the same time, the raw spatial distance separating two locations may not be meaningful: a short walk and a short car trip typically cover very different distances in the same amount of time, and the cognitive and economic costs associated with air travel depend only mildly (if at all) upon distance [17]. Modeling frameworks such as the Theory of Intervening Opportunities [18] and the recently introduced Radiation model [19] further argue that raw distances are not necessarily the most effective determinant for travel. In this work we show the importance of incorporating how frequently an individual travels between two locations, which naturally accounts for spatial and dynamic effects while revealing the underlying spatiotemporal features of human mobility.

## Results

**Figure 1:**
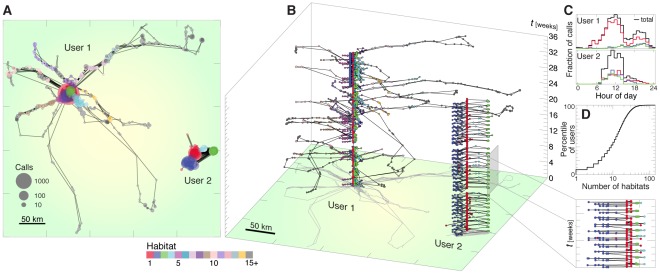
Habitats reveal the spatiotemporal substructure of human mobility patterns. (**A**) Spatial trajectories of two users, one traveling to a large number of locations and another covering a smaller range. Node size indicates the amount of time spent at a particular location (as quantified by mobile phone activity), node color represents the location's habitat detected using Infomap (see Methods), and line width approximates the number of trips between locations. Habitats are ordered by call volume such that Habitat 1 contains the most calls. (**B**) Exploding the spatial trajectories from A in time (vertical axis), the recurrent nature of human mobility becomes evident, with a number of trips featuring both consistent destinations and consistently repetitive occurrence (zoom). These features are the root cause of the high predictability that human motion is known to possess. (**C**) The daily call dynamics of the three most active habitats, as well as the overall dynamics (summed over all habitats). The primary habitat contains the majority of temporal activity. We see that User 1 tends to occupy his or her second and third habitats primarily at night, while User 2 is more evenly distributed. (**D**) The distribution of the number of habitats per user. The median number of habitats is 11. Due to their typical heterogeneity, we characterize population distributions using percentiles, proportional to the cumulative distribution.

Beginning from a country-wide mobile phone dataset [20], [7], [21], [8], [15], [22], [23], [24], we extract 34 weeks of call activity for a sample population of approximately 90 thousand phone users. Each call activity time series encodes the spatiotemporal trajectory of that user. (See Materials and Methods and File S1 for details about the data.) For each user we construct a directed, weighted *mobility network* capturing the detailed flows between individual locations (represented using cellular towers). Examples of both mobility networks and spatiotemporal mobility flows are shown in Figs. 1A and B, respectively. The recurrent and repetitive nature of human motion is clearly visible in Fig. 0B, where we explode the user trajectories vertically in time. We apply to each user's mobility network an information-theoretic graph partitioning method known as Infomap [25], which uses the flows of random walkers to find groups of dynamically related nodes in directed, weighted networks. We do not use spatial or distance information in partitioning, instead Infomap mirrors the stochastic process underlying human mobility flows; see File S1 Sec. S3 for details. (Infomap's underlying mechanism is further justified in this context by the results of [22].) The groups of locations that we discover, which we refer to as mobility “habitats,” will be shown to be crucial to both the spatiotemporal dynamics of human motion, and to the interplay between mobility and human interaction patterns. We rank habitats in decreasing order of phone activity, such that a user's most frequently visited habitat is Habitat 1 or the primary habitat. We observe that human mobility is almost universally dominated by the primary habitat, where the majority of user call activity occurs–and thus it incorporates both home and work, home and school, or other major social contexts–along with a number of less active subsidiary habitats (see Fig. 1C, File S1 Fig. B, Sec. S3.2). We further see in Fig. 1D that most users possess 5–20 habitats, while only approximately 7% of users have a single habitat. Note that these habitats, unique for each member of the population, differ greatly from existing work on partitioning mobility or social connectivity [26], [13], [27], which instead focus entirely on partitioning a single geographic network aggregated from large populations.

### Spatial characteristics

**Figure 2:**
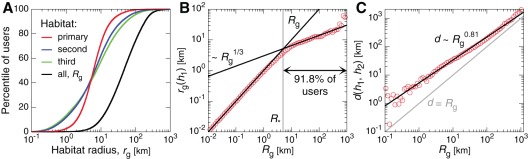
Spatial properties of mobility habitats. We characterize each habitat's spatial extent by computing the radius of gyration 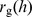 considering only calls placed from locations within habitat 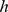. (**A**) The distribution of habitat radii over the population shows that the primary habitat tends to be more spatially compact than the less frequented habitats, though most are consistently smaller than the total 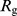 computed using all phone activity. (**B**) The growth in the radius of the primary habitat 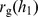 as a function of total radius 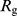. For 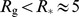 km, we see 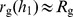, indicating that those users are characterized by a single habitat. In contrast, 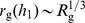 for 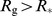. Since approximately 92% of the population have 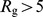 km, the majority of users exist in a regime where their primary habitat encompasses a potentially far smaller spatial region than their total mobility. (**C**) For users with multiple habitats, the distance 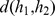 between the first and second habitat's centers of mass is consistently greater than 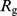 (grey line) and exhibits power law scaling, 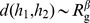, with 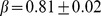. Taken together, we see that most habitats are both well separated and spatially compact, and that the magnitude of 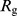 is primarily due to movement between these habitats.

The spatial extent of a user's total mobility pattern has been shown to be well summarized by a single scalar quantity, the radius of gyration, or gyradius, 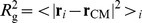, where 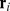 is the spatial position of phone call 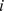 and 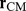 is the user's center of mass [7]. In addition to using the global gyradius we also compute the reduced radius of gyration 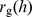 for each habitat 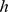, considering only those locations and calls contained within that habitat. In Fig. 2A we plot the population distributions of the first three habitat's 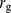, compared with the total gyradius 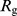 considering all calls placed from all visited locations. This shows that the spatial extent of habitats tends to be far smaller than the total mobility, often by an order of magnitude, and that most users have a habitat 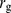 between 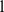
–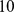 km. See also File S1 Fig. D. In Fig. 2B we study the functional dependence of the primary habitat's gyradius, 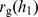, versus 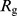. We uncover an intriguing power law scaling relation characterized by two regimes, where 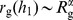 with 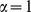 for 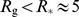 km, and 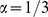 for 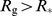. The linear relationship below this critical radius 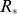 indicates that those users (roughly 8% of the population) are mostly characterized by a single habitat. (In fact, only 54.8% of users with 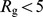 km have one habitat, but that 97.6% of their calls on average occur within their primary habitat.) But once a user's range extends beyond this critical 5 km cutoff (true for 92% of the population) a new regime emerges where multiple habitats exist and tend to be far smaller and more spatially cohesive than the total mobility (since 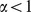). (For users with 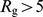 km, only 2.9% have one habitat and the primary habitat accounts for 78.7% of activity on average.) Finally, in Fig. 2C we show the geographic distance 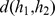 between the centers of mass of the two most heavily occupied habitats, as a function of 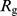. This also exhibits a power law scaling, 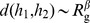 with 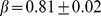. These distances tend to be far larger than the total 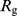 (gray line), indicating that the magnitude of 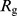 is primarily determined by movement between spatially cohesive and well separated habitats.

### Temporal characteristics

Given the importance of habitats to the spatial extent of human motion, one must ask: how do these habitats form and evolve over time? To what extent are the temporal dynamics of human movement reflected in the evolution of these habitats? Recently, considerable effort has been undertaken to understand the intriguing temporal features of human mobility, including the previously observed ultra-slow growth in time 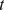 of 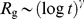, with 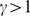 [7], [15]. Given the contribution of habitats to the magnitude of 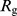, shown in Fig. 2, a primary question becomes: how do habitats impact these temporal features? For example, how do individual habitat 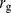
's evolve over time, compared with that of the total 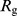
?

**Figure 3:**
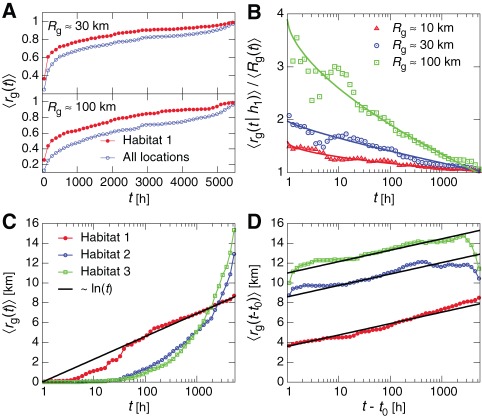
Temporal evolution of human mobility. (**A**) The time evolution of 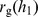 compared with 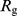, where both are normalized by their final values at the end of the observation window. We see that the primary habitat tends to reach saturation faster than the overall gyradius, indicating different temporal dynamics. (**B**) To quantify the saturation rate, we plot the ratio of the two curves from A, for groups of users with different 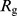. We see that the primary habitat saturates more quickly as the overall 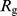 grows. Solid lines of the form 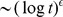 provide a guide for the eye. (**C**) The unnormalized growth in habitat size for the first three habitats. The primary habitat shows a distinct, approximately logarithmic temporal scaling. The other habitats show a longer delay before 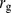 begins to grow polylogarithmically. (**D**) Given this delay, we now shift the time series of 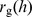 for each habitat by 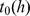, the time when the user first entered habitat 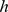. Doing so we recover pure logarithmic scaling for all habitats, 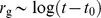, indicating that a major factor in the scaling of human mobility is the delay it takes for a user to transition to his or her non-primary habitats.

In Fig. 3 we study the temporal evolution of 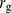 and 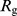 by considering only those calls occurring up to time 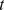, from either individual habitats or all locations, where 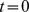 is the time of the user's first call. In Fig. 3A we plot the time series of 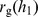 and 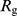, normalized by the final values of each respective series. We observe that 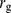 saturates at its final value more quickly than the total mobility's 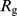. To further quantify this saturation, we plot in Fig. 3B the ratio between 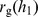 and 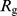 as a function of time, for groups of users with different final values of 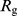. We observe increasingly rapid saturation of 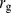 as the total 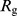 increases. This implies that the primary habitat is explored more quickly than the total extent of a user's mobility pattern and that users who cover large distances explore their primary habitat more quickly relative to their total mobility than users who traverse relatively smaller regions. This is particularly interesting as one may initially expect such exploration to be at a constant rate relative to their total 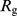. In Fig. 3C we study the temporal evolution of 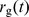 for the first three habitats, averaged over users with 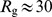 km. We observe approximately logarithmic growth, 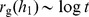, for the primary habitat (slower growth than that observed in [7], [15]) while subsidiary habitats' gyradii 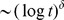, with 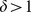 (growth more similar to [7], [15]). However, this analysis neglects an important detail: users do not begin exploring all of their habitats at the same time. Therefore in Fig. 3D we plot the same population-averaged radii, but we now individually shift each user's time series of 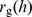 by a time 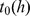, the time the user first entered habitat 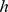, not simply made his or her first global call. Doing so accounts for the waiting times for users to visit habitats within our observation window. With this crucial correction we reveal *for all habitats* purely logarithmic growth in 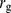, implying a universality in the exploration of habitats (which differ only in their overall spatial scale, with the primary habitat tending to be the most compact). Thus, the polylogarithmic growth of 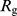, where 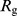 is initially small then grows faster than logarithmic in time, is primarily due to the temporal delay it takes users to exit their respective primary habitats and then rapidly traverse a relatively large distance to reach their other habitats. We further study these habitat entrance times in File S1, Sec. S3.2 and Fig. E.

### Social characteristics

**Figure 4:**
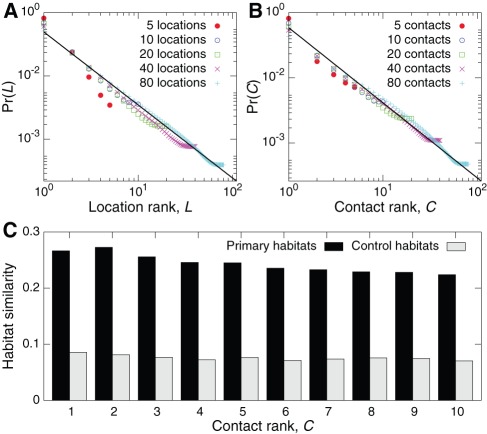
Contact activity and habitats. (**A**) The Zipf law governing the probability 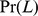 for a user to visit his or her 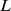-th most visited location, as first observed by González et al. [7]. The solid line indicates 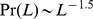 (**B**) Interestingly, we observe an identical Zipf law for the probability 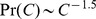 for a user to call his or her 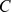-th most contacted partner. This holds regardless of the total number of contacts for a user. This implies that the same underlying mechanism may govern how users choose both locations to visit and friends to contact. (**C**) The habitat similarity, related to the number of common locations, between a user's primary habitat and the primary habitat of their contacts, averaged over pairs where both users are present in our data. We see that, despite the Zipf law in B, user's habitats tend to be surprisingly similar to their most contacted ties, even for those less frequently contacted users. Control habitats, generated by randomly shuffling a user's visited locations between his or her original habitats, exhibit lower similarity. See Methods for habitat similarity and controls.

Finally, a major question in the realm of mobility and human dynamics is the connection between spatiotemporal dynamics and other activity patterns [9]. For example, information spreading in heterogeneous systems of agents is a process that involves both the spatiotemporal mobility of the agents and their long-range communication activities. In this context, would the currently occupied habitat affect or be affected by how a user chooses a particular communication partner to engage? Such questions can also be addressed with mobile phone data, where phone communications capture a primary mode of information diffusion on the underlying social network [20]. To begin, we first recall a result from González et al. [7]. They found that users occupy locations following a Zipf law, where the probability 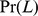 to visit the 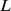-th most frequented location follows 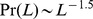. We reproduce this result in Fig. 4A. Interestingly, we discover (Fig. 4B) a potentially identical mechanism for how users choose to contact their communication partners, i.e. the probability 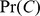 for a user to call his or her 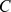-th most contacted partner also follows 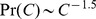. See also [28]. Finally, a number of users within our population have contacts that are also within the population, meaning we have habitat information for both users. An interesting question is: how similar are the habitats of users in close communication, and will this similarity be lower for pairs with less frequent interaction? We measure the similarity between the primary habitats of pairs of users interacting with one another by computing the relative number of locations the habitats have in common (see Methods and materials). In Fig. 4C we plot this Habitat similarity as a function of contact rank 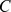. We see that, despite the Zipf law in Fig. 4B, users' primary habitats tend to be highly similar to the primary habitats of their most contacted ties. Nevertheless, there is little dependence on contact rank: one partner that is contacted an order of magnitude less often than another has almost the same primary habitat similarity. In other words, it takes very little communication to generate considerable habitat overlap [10]. Meanwhile, control habitats, generated by randomly distributing each user's visited locations between their habitats (see Methods and materials), show smaller similarity and no effective trend.

**Figure 5:**
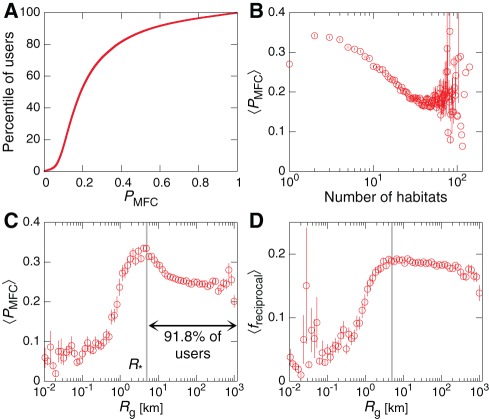
Communication and mobility dynamics. We characterize the interaction concentration of a user by 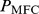, the probability for that user to place a call to his or her Most Frequent Contact. (**A**) The distribution of 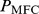 over the population shows that most users have 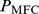 between approximately 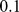 and 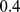. (See also Fig. 4b.) (**B**) To connect the concentration with user mobility, we study how the mean 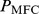 varies with the number of habitats each user possesses. We see that 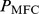 gradually decays as the number of habitats grows, indicating that broadly traveled individuals tend to more evenly distribute their calls over their partners. (**C**) Studying 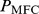 as a function of 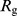, we uncover an intriguing relationship. For users with particularly small mobility ranges, 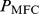 is small but grows as 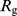 grows. This continues until 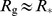, the same critical radius size observed in Fig. 2b. The mean 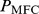 then decays for 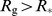. Surprisingly, this implies that the distribution of call activity over a user's partners exhibits different behavior depending on whether that user possess one mobility habitat, or many habitats. (**D**) The fraction of reciprocated contacts 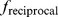 as a function of 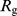 shows a trend similar to 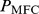. Not only do those users with small 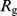 tend to be distinctly less socially concentrated compared with most users, they also tend to make more non-reciprocated contacts (see File S1 Fig. C for details). Error bars indicate 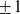 s.e.

We further characterize the “interaction concentration” of a user by introducing 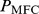, the probability that the next call placed by the user goes to that user's Most Frequent Contact, the partner that is most often in contact with the user. Users with a small 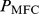 tend to distribute their calling activity more evenly across their partners, while users with large 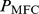 are more concentrated and focus much of their attention upon a single individual. In Fig. 5 we study how 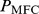 depends on the properties of a user's mobility pattern. First, in Fig. 5A we show the distribution of 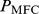 over the user population. Most users possess 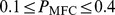 while very few users have either very small or very large 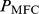. In Fig. 2B we connect this interaction concentration with the user mobility patterns by showing that the mean 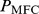 decays as the number of habitats a user visits grows. This means that users who travel broadly, leading to complex mobility patterns and multiple habitats, tend to distribute their communication activity more uniformly over their contacts. Next, in Fig. 5C we quantify how 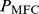 varies with the total gyradius 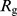. We see an intriguing connection to a previous result: For users with small 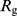, the 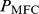 is small but grows as 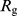 grows. This continues until 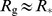, the same critical radius that appeared in Fig. 2B. Above 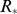, we see that 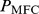 now slowly decays with 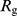. To further understand this, we plot in Fig. 5D the fraction of reciprocated contacts 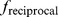 (see Materials and Methods) as a function of 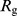. The plot exhibits a roughly similar trend as Fig. 5C: 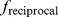 grows while 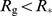 then, above the same critical radius, 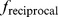 decays slowly with 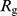. This decay relative to the peak value at 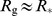 is slower for 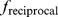 than for 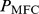.

Taken together, Figs. 5C and D show that when 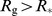, user communication activity–both how much they concentrate upon their MFCs and how many of their ties are reciprocated–depends only weakly on 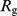. However, those users with low 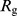 tend to show distinctly different behavior, both being less concentrated on their MFCs compared with most users, and making a larger number of non-reciprocated contacts (File S1 Fig. C). Since users with 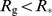 primarily possess a single habitat, these results imply that the mechanism governing how users distribute their activity over their contacted partners may differ for those users with a single habitat compared with those users whose mobility leads to multiple habitats. We used Kendall rank correlation and associated hypothesis tests [29] to verify the statistical validity of the observed relationships. See File S1 Sec. S6 and Table A for hypothesis tests between these and additional measures.

The mobile phone data also provides demographic information for the majority of users, specifically self-reported age and gender. In File S1 Sec. S4 we study the results of Fig. 5 after decomposing the sample into age and gender groups. One may expect these results to change when focusing on these different groups. Yet in Figs. F and G we find qualitatively similar results to Fig. 5 with only small differences: 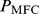 tends to be slightly higher for women than for men, and increases with user age. After considering these demographic dependencies on 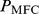, we observe the same relationships between communication activity and mobility.

## Discussion

We have shown that accurately understanding human mobility requires an analysis using the complete spatiotemporal flows captured for each user. Basic measures such as the gyradius 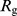 constitute an excellent starting point, but such single scalar quantities simply cannot capture the full complexity of an individual mobility pattern. As the quality and quantity of available data increases, we expect our understanding of the various factors shaping human mobility to continue to improve.

Given that users spend the majority of time occupying their primary habitats, understanding the detailed features of the primary habitat will be crucial for applications such as search and rescue during emergencies [23] or containing the spread of epidemic diseases [3], [4], [5], since most users will be within their primary habitats at the onset of such events. Meanwhile, detailed information regarding unusual trips away from the primary habitat may prove useful both for curtailing diseases and for optimizing transportation infrastructure and energy usage. Likewise, the universal logarithmic scaling laws for intra-habitat mobility uncovered in Fig. 3D are not accounted for by current modeling frameworks [15]; more effort may be necessary to acceptably model the microscopic structure of individual human motion. The connections we reveal between communication dynamics and human mobility may have important consequences for understanding the spread of information or rumors through a population, as such processes may spread both spatially and socially [30]. Further investigation of such connections may prove fruitful in a number of areas, including information diffusion and social contagion.

## Materials and Methods

### Dataset

We use a large-scale, de-identified mobile phone dataset, previously studied in [20], [7], [21], [8], [15], [22], [23]. We sample approximately 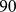 thousand users from the total dataset, according to the activity criteria introduced in [8] (see also File S1 Fig. H). We retain nine months of phone activity for each user. A “call” is either a text message or a voice call, and we use the cellular tower that handled the communication to represent the location 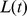 of a call made at time 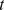. Call times are kept at an hourly resolution. The coordinates of these towers are used to compute the radii of gyration [7]. Phone call recipients determine the communication partners of a user. Since a single phone call between two individuals may not represent a meaningful tie, we consider user 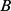 to be a partner of user 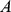 only if we observe at least one reciprocated pair of calls (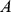 called 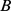 and 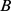 called 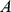) [20]. We do not require user 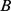 to be in our sample population, except when we compute habitat similarity. We define the fraction of reciprocal ties for user 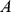 as 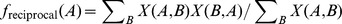 where 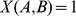 if 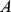 contacted 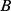 at least once, and zero otherwise.

### Finding mobility habitats

For each user we convert their trajectory 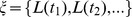, with 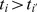 for 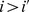, into a weighted digraph where the weight on link 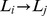 represents the number of times the ordered pair of locations 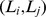 was observed in 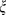 (File S1 Fig. A). The community discovery method Infomap [25] was applied to each digraph, using the default parameters (10 attempts and self-loops ignored). The discovered groups of locations are the habitats for that user. Habitats are ranked by total number of calls.

### Habitat similarity

For a user 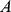 with contact 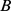, both present in our sample, we define the similarity 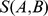 between their primary habitats to be the Jaccard coefficient between the sets of locations comprising those habitats. If these sets are disjoint 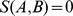, whereas 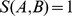 if they overlap completely.

### Controls

It is important to understand the significance of the results we have presented here, in particular whether the results associated with the habitats we find are meaningful. We compute null or control habitats, generated for each user, by randomly assigning that user's visited locations to habitats while preserving the number of habitats and the number of locations within each habitat. This strictly controls for the habitat size distributions while testing the effects of habitat membership. In File S1 Fig. I we further show that the pure logarithmic time evolution is absent in control habitats, indicating that the temporal evolution we observe in Fig. 3D is not due to the relative sizes (numbers of locations) of the habitats, nor to simply the number of habitats, but due more fundamentally to their spatial structure and the spatiotemporal flows of the users. In Fig. 1 we see that these control habitats have lower similarity than the actual habitats. See File S1 Sec. S6 for details.

## Supporting Information

File S1 **Supplementary text and figures.**
(PDF)
