# Supplementary material for: Mesoscopic Structure and Social Aspects of Human Mobility
Source: PLoS One. 2012 May 31;7(5):e37676. doi: 10.1371/journal.pone.0037676 (PMC3365118; doi:10.1371/journal.pone.0037676)
Supplement: File S1 — Supplementary text and figures. (PDF) [file pone.0037676.s001.pdf]

Supporting Information

Mesoscopic structure and social aspects of human mobility
by James P. Bagrow and Yu-Ru Lin

Table of Contents

S1 Dataset **1**

S2 Mobility networks **2**

S3 Mobility habitats **2**
S3.1 Justification for Infomap 3
S3.2 Additional properties of mobility habitats 3

S4 Demographic and communication effects **6**

S5 Data sparsity **7**

S6 Controls and hypothesis tests **9**

References **10**

List of Figures

A Properties of mobility networks. 3
B Additional habitat statistics 4
C More habitat properties 5
D Habitat gyradius versus habitat rank 6
E Population distributions of habitat arrival times 6
F Demographics and extensions of interaction concentration 7
G Demographics and tie reciprocity 8
H Missing data do not affect most statistics 8
I Temporal evolution of gyradius for real and null habitats 9

List of Tables

A Nonparametric correlations between mobility and communication 10

S1 Dataset

We use a set of de-identified billing records from a Western European mobile phone service provider [1, 2, 3, 4, 5, 6]. The records cover approximately 10M subscribers within a single country over 3 years of activity. Each billing record, for voice and text services, contains the unique identifiers of the caller placing the call and the callee receiving the call; an identifier for the cellular antenna (tower) that handled the call; and the date and time when the call was placed. Coupled with a dataset describing the locations (latitude and longitude) of cellular towers, we have the approximate location of the caller when placing the call. For

this work we do not distinguish between voice calls and text messages, and refer to either communication type as a "call."

These phone records cover approximately 20% of the country's mobile phone market. However, we also possess identification numbers for phones that are outside the service provider but that make or receive calls to users within the company. While we do not possess any other information about these lines, nor anything about their users or calls that are made to other numbers outside the service provider, we do have records pertaining to all calls placed to or from those ID numbers involving subscribers covered by our dataset. Thus egocentric networks [7] between users within the company and their immediate neighbors only are complete. This information was used to generate egocentric communication networks and to study the MFC probability and its relationship to human mobility patterns.

We generate a sample population of approximately 90k users (specifically, $N = 88137$), using the criteria introduced in [3]. Each user's call history during our nine-month tracking period yields three time series: (i) event times for when calls are made, kept to an hourly resolution; (ii) locations of calls, as quantified by the cellular tower transmitting the call; and (iii) communication partners who receive calls. These time series allow us to reconstruct both geographic trajectories and egocentric communication networks for each user.

S2 Mobility networks

We construct for each sample user a weighted, directed mobility network G (or *MobNet* for short) using the user's time-ordered trajectory $\{L(t_1), L(t_2), \ldots\}$, where $L(t)$ is the location the user called from at time t. Each link $(L_i \to L_j)$ in G represents the user placing a call at location L_i followed by a call at location L_j. The weight on link $(L_i \to L_j)$ gives the number of times the user made that particular relocation during the sample window.

In Fig. Aa we draw the mobility network of a single user. This network was drawn using a typical force-directed graph layout algorithm [8] and does not use geographic tower locations. We see several dense cores comprising groups of frequently visited locations as well as a number of long loops or chains representing sequential calls placed during one-time, typically long-range trips. These mobility networks feature broad degree distributions, well described by a truncated power law of the form $\Pr(k) \sim k^{-\beta} e^{-k/\kappa}$, for constants β and κ, where k is the number of connections of a location (or number of unique locations visited before or after visiting that location). The nature of this broad distribution is not surprising given the Zipf law for location selections, observed in [2] (see also Main text Fig. 4a). Although one may not expect this distribution to hold for both in- and out-degrees, we do observe similar patterns in our directed networks.

S3 Mobility habitats

In this work we start by identifying groups of related locations, for each user. These groups may correspond to home, work, school, or any number of other contexts throughout daily life. (In practice, we find that the most occupied habitat accounts for the majority of activity and thus must be both home and work, home and school, etc.) The mobility networks we study are inherently spatial, possessing a unique geographical embedding, yet we do not discover these groups through spatial clustering methods, so it may be misleading to refer to these groups as clusters. Likewise, although we use a community detection method known as Infomap [9] to find these groups, mobility networks are not social networks, so referring to these groups as communities may also be misleading. To avoid confusion, we instead term these groups "habitats." We rank each user's habitats by the total number of phone calls that occur within the habitat, so that Habitat 1 is most

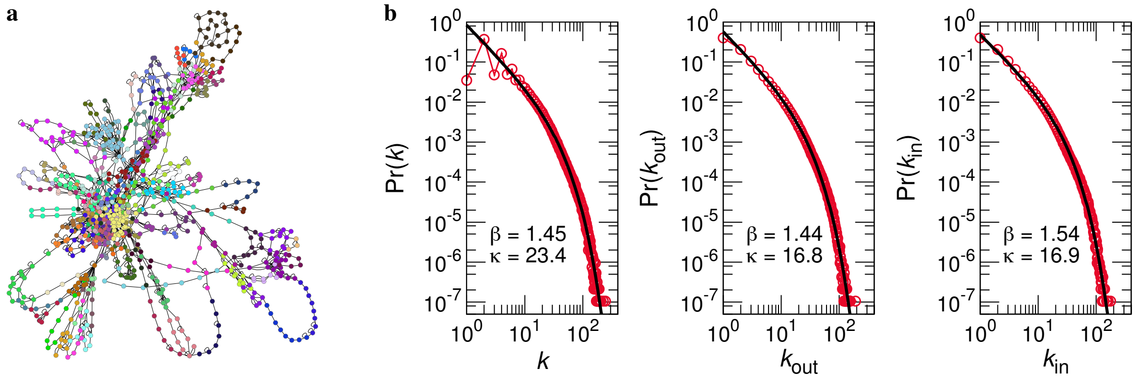

Figure A: Properties of mobility networks. **(a)** An example mobility network (MobNet), drawn without using spatial coordinates. Several dense cores are visible, as are a number of unusually long loops, representing one-time trips. Node colors indicate habitats, discovered using Infomap [9]. Link weights and directions have been omitted for clarity. **(b)** Degree distributions (undirected, outdegree, and indegree) for all MobNets. All are well described by a power law with an exponential cutoff, $\Pr(k) \sim k^{-\beta} e^{-k/\kappa}$, meaning that users typically visit many locations only a small number of times while a few locations are visited many times. Note that here "degree" refers to the number of connections per location (the number of unique locations visited before or after visiting that location) not the number of communication partners a user has.

active, Habitat 2 is second most active, etc. Habitats are not ranked by number of locations, though these tend to correlate. See Methods and materials in the main text for details on how to apply Infomap.

S3.1 Justification for Infomap

There are numerous algorithms for detecting communities in networks [10]. We believe Infomap to be ideally suited for our purposes here, for two main reasons. The first reason is that it is specifically capable of handling both weighted and directed networks, and much of the focus of the original publication [9] was devoted to the sometimes confusing effects of community structure in directed networks. Infomap's theoretical basis rests upon the notion of random walkers moving through the network. These walkers can readily adapt to link weights and link directions, no modifications to the underlying algorithm are necessary.

The second reason for adopting Infomap relates to a result from Park, Lee, and González [11]. They present the at-first-glance paradoxical result that a random walk model on an empirically-derived mobility network does well at reproducing macroscopic phenomena such as the gyradius. This seems surprising given that human beings have long-term memory, and consistently travel between fixed sets of destinations [2], unlike a diffusing random walker. Even in [3] it was shown, using estimates of the Kolmogorov entropy of a human trajectory, that there is more information in a trajectory than estimated by the Shannon entropy alone. (The resolution of this paradox is to note that a random walker exploring a new space of locations will not be able to *generate* mobility networks such as those we derive from the mobile phone data; a more complex modeling framework is necessary [4].) Thus, while there is information in a human trajectory beyond the mobility network, the mobility network still captures a great deal of mobility phenomena. Infomap's theoretical basis exactly matches this random-walker-on-a-mobility-network model.

S3.2 Additional properties of mobility habitats

We remark on several additional features of mobility habitats not fully discussed in the main text. In Fig. B we plot the distributions over the sampled user population of several quantities of interest for the habitats.

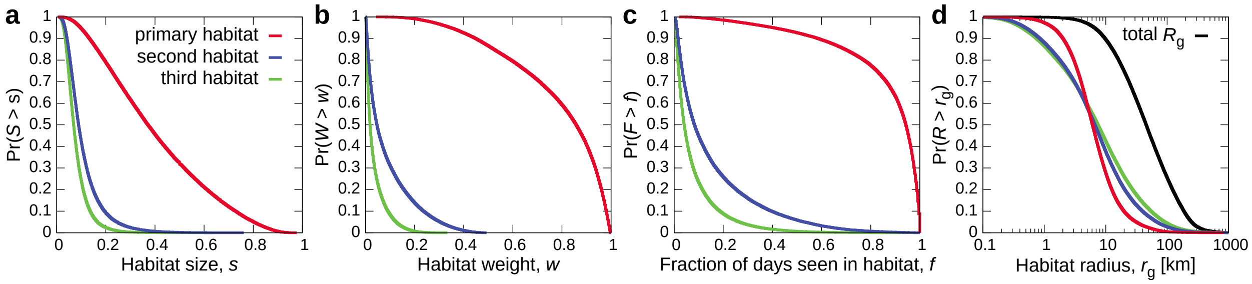

Figure B: Additional habitat statistics. We plot the complementary cumulative distributions of habitat size (fraction of unique locations within the habitat), habitat weight (fraction of calls placed from within the habitat), the fraction of days during the nine-month window where the user was active and was observed in the habitat, and the habitat radius of gyration. The gyradii distributions were also shown in main text Fig. 2a. We see that the primary habitat occupies most locations, the majority of phone activity, and that most users are found in the habitat nearly every day. Thus the primary habitat captures the majority of user dynamics.

These are the "size" of each habitat, as given by the number of unique locations within the habitat; the "weight" of each habitat, given by the fraction of phone calls placed from locations within the habitat; the fraction of days during the nine-month sample window where the user was active making calls and observed in the habitat; and the distribution of habitat spatial extent, given by the gyradius. We see that the primary habitat tends to contain most locations and the overwhelming majority of call activity, and that many users appear in their primary habitat almost every day. Thus the primary habitat alone tends to capture the intrinsic or typical day-to-day activity of a user.

We explore more relationships between habitats, spatial features, and call activity in Fig. C. We begin in Fig. Ca by plotting the distribution of the average number of locations per habitat. This distribution is very sharply peaked around 7–8 locations per habitat, implying a typical habitat size (in numbers of locations, not spatial extent). In Fig. Cb we consider the average number of calls (either voice or text) placed by users as a function of their total number of habitats. We see a slow increasing trend.

Meanwhile, a number of user features can be related to the total gyradius R_g. In Fig. Cc we study habitat weight vs. R_g. We see that this weight is approximately 1 until $R_g \approx R_*$, after which it slowly decays to around 0.5, on average. This means that users tend to be less concentrated within their main habitat as their movements grow, which makes sense. We return to the distribution of R_g in Fig. Cd but now consider it conditioned on users having a specific number of habitats total. We see that users with a single habitat tend to have far smaller R_g than users with more than one habitat, which is reasonable, while users with three habitats have a similar R_g distribution as users with only two habitats. A related quantity is the average number of habitats as a function of R_g, shown in Fig. Ce. This number is essentially flat at one habitat until $R_g \approx 1$ km, after which it slowly grows with R_g.

It is especially interesting to compare social activity with R_g, as was done in main text Fig. 5. Doing so further sheds light on some of the main text results regarding P_{MFC} and $f_{\mathrm{reciprocal}}$, the fraction of ties that are reciprocated. First a question of data sparsity. It seems reasonable that users with very low R_g may simply not use their mobile phones much. In Fig. Cf we show that this is not the case by computing the average number of calls as a function of R_g: users with low R_g actually tend to make more calls and this call volume slowly decays as R_g grows. Perhaps this means that low R_g users have more social contacts than users with higher R_g? To see this we plot in Fig. Cg the number of unique call recipients as a function of R_g and we see a rather dramatic increase in these ties at or slightly before $R_g \approx R_*$. This means that users tend to have much more diverse calling patterns when they seldom move, although we caution that the statistics are not

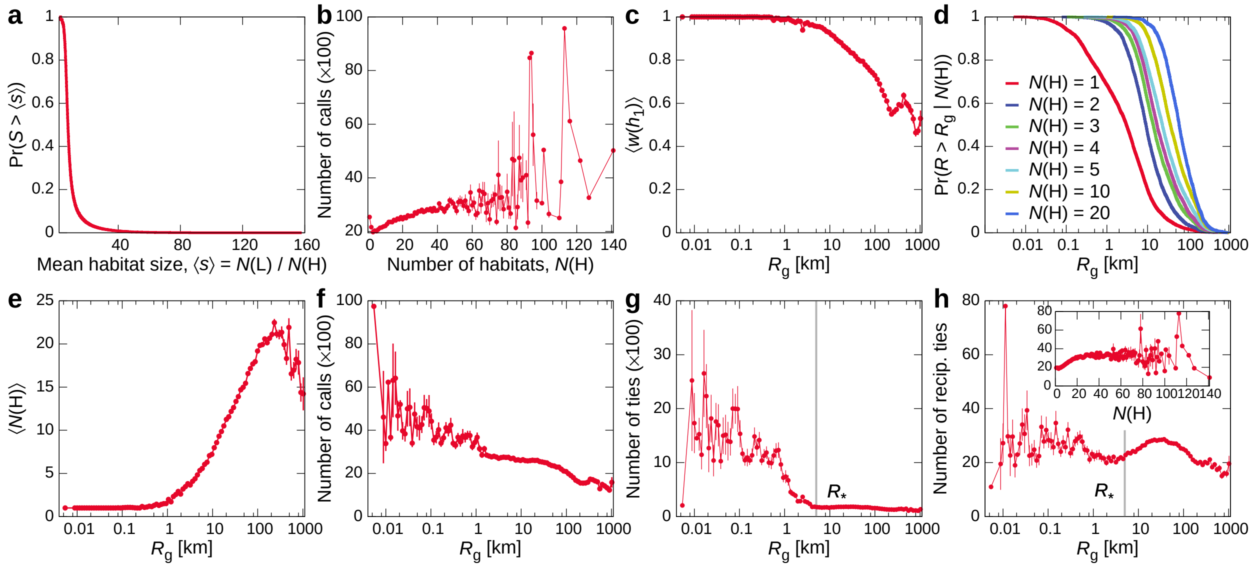

Figure C: More habitat properties. (**a**) The distribution of the average number of locations per habitat is narrowly peaked around 7–8 locations per habitat, with only a handful of habitats containing large numbers of locations. (**b**) The total number of calls placed by users as a function of the number of habitats. There is a weak increasing trend. (**c**) The fraction of call activity $w(h_1)$ occurring in the primary habitat as a function of R_g. The majority of activity occurs within the primary habitat. This slowly drops as R_g grows, though the trend is weak. (**d**) The distribution of R_g for users with a given number of habitats $N(H)$. We see that users with a single habitat tend to have smaller total R_g than users with multiple habitats, whereas the distributions of R_g change less for users with multiple habitats as the number of habitats increases. (**e**) The average number of habitats vs. R_g. Compare this with main text Fig. 2B. (**f**) The average total number of calls shows a slight, relatively constant downward trend with R_g. (**g**) The average number of ties vs. R_g. We see that users with small R_g show a sharp increase in the number ties, an effect stronger than the increase in the number of calls shown in f. (**h**) The average number of reciprocated ties vs. R_g. We see no consistent trend as R_g varies. This means that the increase in the fraction of reciprocated ties for users with $R_g > R_*$, shown in main text Fig. 5D, is due to the decrease in the number of non-reciprocated ties as R_g increases, show here in g. (Inset) The number of reciprocated ties as a function of the number of habitats $N(H)$. We see a dependence for $N(H) < 20$. Error bars represent ±1 s.e.

plentiful at the extreme range of R_g. Likewise, an important fact is that these ties may not all be meaningful. As mentioned in the Materials and Methods section of the main text, we estimate a tie to be meaningful if it is reciprocated. In Fig. Ch we see that the average number of reciprocated ties is approximately independent of R_g, implying that, while many different activity features are affected by mobility phenomena, the number of meaningful social ties may be constant regardless. (On the other hand, we do see a weak dependence on the number of habitats, see inset.) The famous Dunbar's number [12] fixing the maximum size of one's social circle is a relevant quantity of interest.

Another spatial feature we study here is the spatial extent of habitat h, captured by its gyradius $r_g(h)$, as a function of the rank h of the habitat. We show in Fig. D that more active (lower h) habitats tend to be smaller on average, going from $r_g \approx 10$ km to $r_g \approx 20$ km as h goes from 1 to 3. However, we see a rapid saturation in r_g to values typically between 25 to 30 km, for $h > 5$. This indicates that there is an **intrinsic upper bound** on effective habitat spatial extent, further emphasizing their cohesive nature.

Finally, we also study the distribution of habitat entrance times $t_0(h)$, the time it takes for the user to first enter a location within habitat h ($t = 0$ is the time of the user's first call). In the main text we show how the delay in entering habitats greatly alters the temporal scaling in r_g, so that habitats grow only logarithmically in time, distinct from the polylogarithmic growth reported in the literature for the full mobility pattern [2, 4]. We present in Fig. E the complementary cumulative distributions of t_0 for the first three habitats, as well as

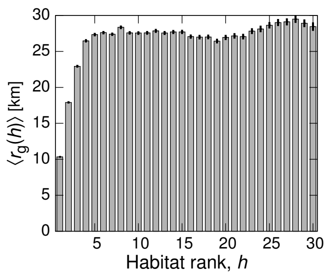

Figure D: Habitat gyradius versus habitat rank. We see that the most active habitats tend to be more compact spatially, on average, than those less frequented habitats. The overall habitat size quickly saturates at around 25 − 30km, however, indicating an intrinsic maximum spatial extent. Error bars represent ±1 s.e.

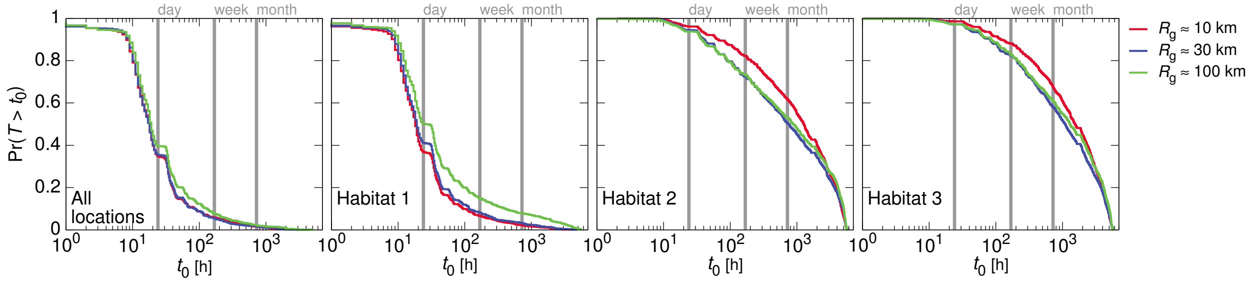

Figure E: Population distributions of habitat arrival times. The distributions of times t_0 when each user placed his or her first call from any location or from within a habitat. Different curves represent groups of users with different total gyradius R_g. We see that most users place their first call within Habitat 1 very early, often within a day of the start of data collection. As R_g grows, these distributions change only slightly, with far ranging travelers having a slightly higher probability of delaying their first appearance in Habitat 1 (green curve). Habitats 2 and 3 show longer waits until users arrive at these locations. Interestingly, there is only a minor dependence on R_g: users with smaller values of R_g (red curve) tend to wait slightly longer to enter these habitats.

the time it takes for the first call to occur (which can be thought of as t_0 for all locations). We see that the distribution for Habitat 1 is functionally similar to the total distribution, while t_0 for Habitats 2 and 3 tends to be higher in value. We see a minor dependence on the total mobility extent, quantified with R_g: Users with higher R_g tend to wait longer before entering Habitat 1, perhaps because they are more likely to be traveling far from home when data collection begins, while users with lower R_g tend to wait slightly longer before entering Habitats 2 or 3, perhaps because they tend to travel less frequently. These results further emphasize the fundamental role that mobility habitats play in determining the magnitude and dynamics of human mobility and travel patterns.

S4 Demographic and communication effects

We decompose the sample population by self-reported age and gender, and present the results from main text Fig. 5 with respect to different age and gender groups. Results for the different groups are shown in Fig. Fa and Fb. We see the same overall features for these groups, with some small quantitative differences, that we observed in the main text for the entire population. We also compare in Fig. Fc the probability of calling the most frequent contact (P_{MFC}) with the cumulative probabilities of calling the top-two and top-three most frequently contacted communication partners. We see that the cumulative probabilities exhibit a similar trend as P_{MFC} with respect to mobility, suggesting that the relationship between mobility patterns and interaction concentration captured by P_{MFC} is stable over the most frequently contacted partners. Finally, in Fig. G we study the fraction of ties that are reciprocated $f_{reciprocal}$ for different age and gender groups. We see a small increase in $f_{reciprocal}$ for users under the age of 30, compared with older users, while there is no

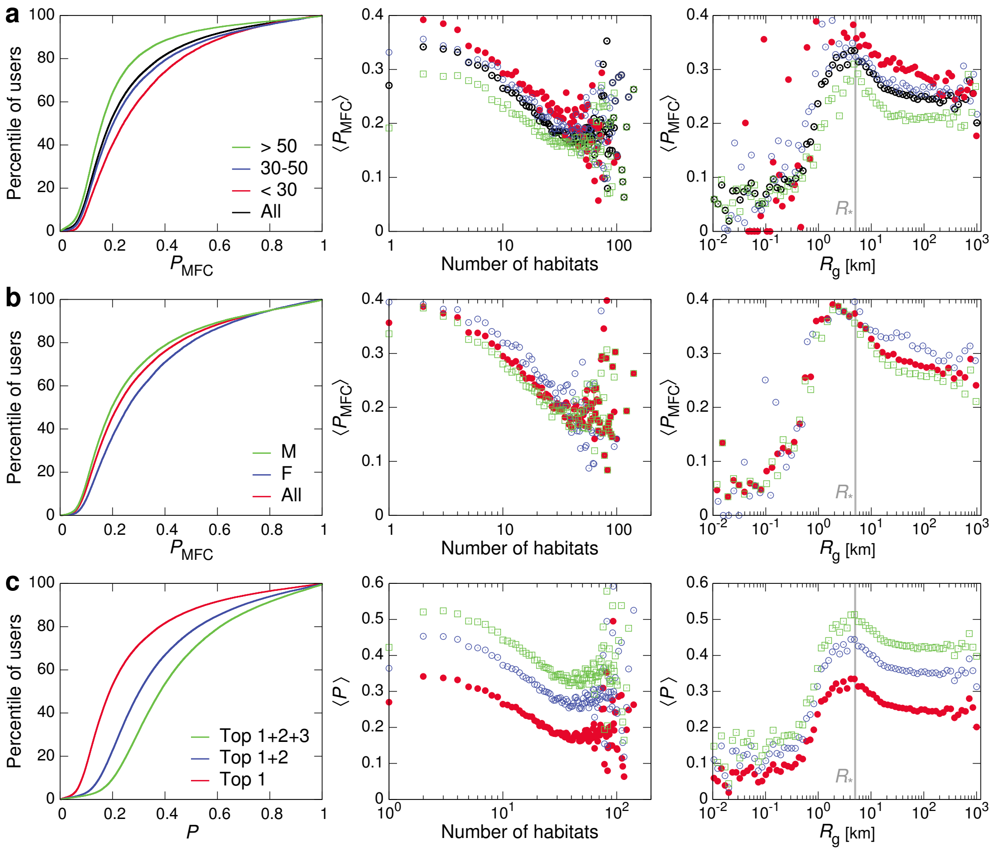

Figure F: Demographics and extensions of interaction concentration. (**a**) Age groups. Users older than fifty tend to be less concentrated on their MFC, while younger generations focus more on their MFC. ("All" indicates all users with self-reported age.) (**b**) Gender groups. Female users call slightly more to their MFC than male users. ("All" indicates all users with self-reported gender.) (**c**) The cumulative probabilities for calling the top-two and top-three most frequently contacted friends exhibit similar trends as P_{MFC} with respect to the mobility measures. Overall, the primary difference for the demographic groups is the average value of their respective P_{MFC}. After accounting for this, we observe the same relationships between P_{MFC} and mobility.

dependence on gender.

S5 Data sparsity

There is an important factor to consider when using mobile phone data and that is how phone usage affects measurements, as data are available only when users engage their mobile phones. While we select active users using the criteria of [3], specifically intended to mitigate such problems, there still exist many time periods where a user does not use the phone and thus we do not have any information. We now study this in further detail.

We compute for each user the fraction of hours q, out of the nine-month window, where the user is not active. In Fig. H we plot the distribution of q over the 90k users; we see that users are inactive on average around 75% of the time. (This distribution, and its consequences, was also discussed in [3].) While this may seem problematic at first, we are able to proceed because we are integrating over such a long time window, extracting robust amounts of data for the quantities we are interested in. For example, in Fig. H we also plot

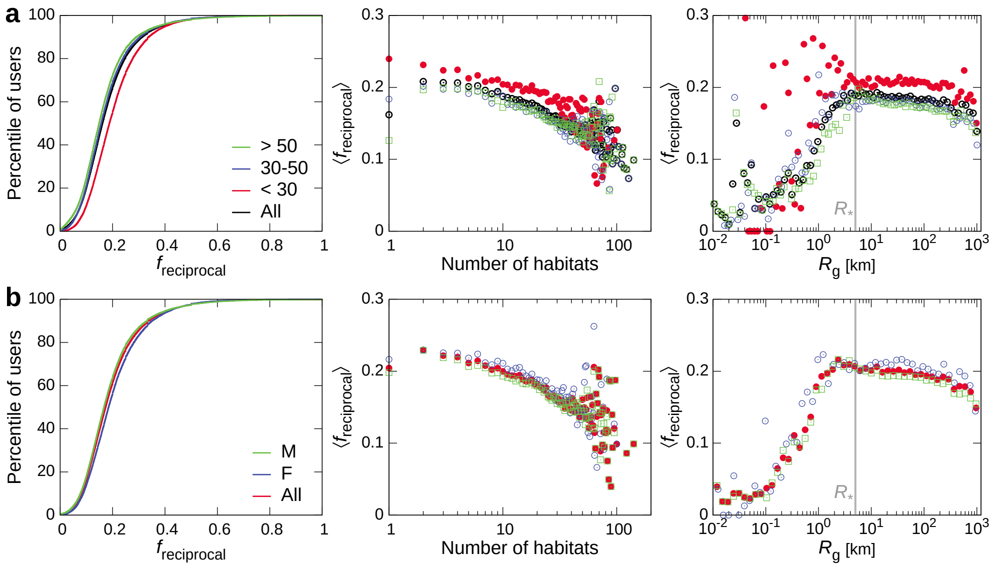

Figure G: Demographics and tie reciprocity. (**a**) Age groups. Users under the age of 30 tend to have slightly more reciprocated ties than older users. (**b**) Gender groups. The fraction of reciprocated ties shows no dependence on user gender.

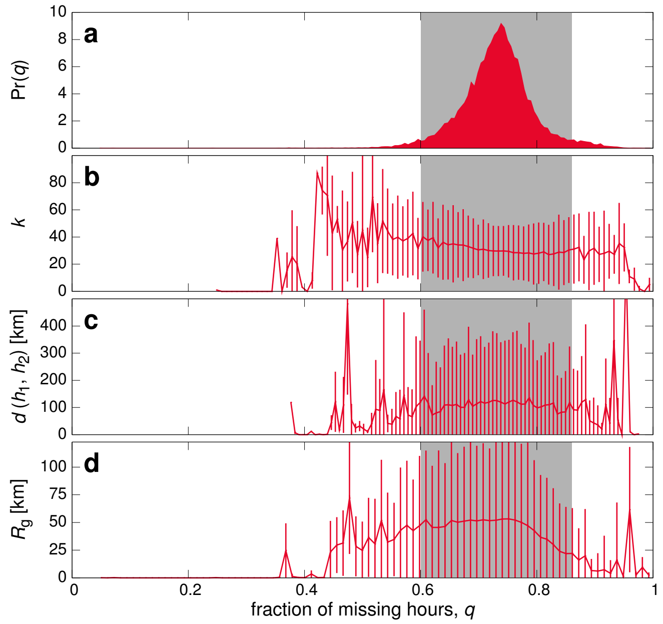

Figure H: Missing data do not affect most statistics. Mobile phone data is inherently incomplete since data is only available when users place calls. We apply the selection criteria used in [3] to mitigate the missing data issue. When considering the fraction of hours q without data out of the 34 weeks, we see that users are typically missing data $\approx 75\%$ of the time (**a**). One may expect this to cause bias, yet for a number of measures (**b**–**d**) we see almost no trend in the shaded region (containing $\approx 95\%$ of the population). Only for the gyradius (**d**) do we see a minor dependence for higher values of q. These results indicate that our measures are not sensitive to missing data. Error bars indicate ± 1 s.d.

the number of communication partners k, the distance between the first and second habitats $d(h_1, h_2)$, and the total gyradius R_g, all as a function of the missing fraction q. We see almost no trend or dependence on q. Meaning that users missing data 40% of the time give the same or similar statistics as users missing data 80% of the time, for example. Only for the gyradius do we see a small drop in R_g for larger values of q. We note, however, that even this trend remains within 1 s.d. and is thus not significant.

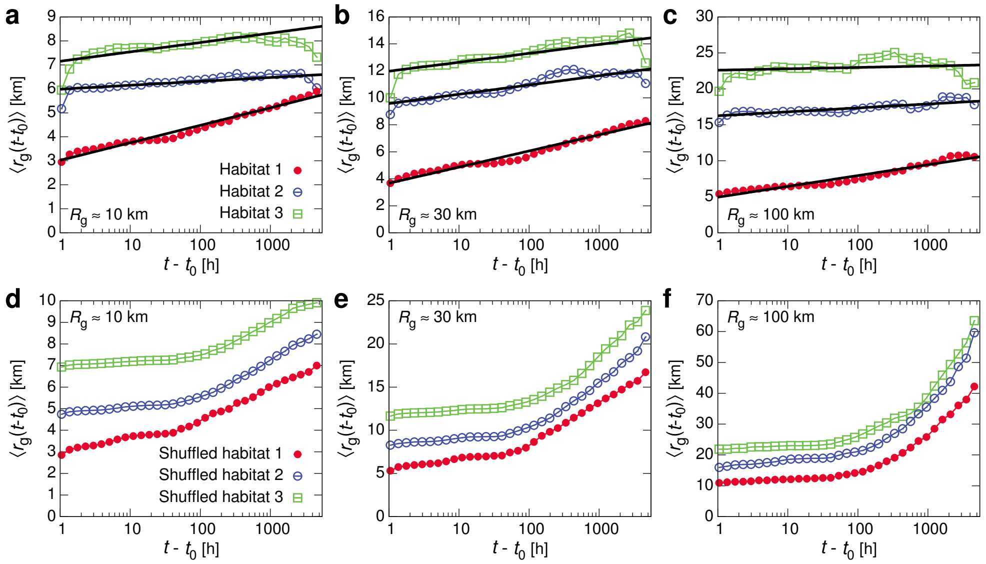

Figure I: Temporal evolution of gyradius for real and null habitats. (a-c) Real habitats. The temporal evolution of $\left\langle r_g(h) \right\rangle \sim \log(t - t_0)$ in all cases for user groups with total $R_g \approx 10$, 30, and 100 km. Straight lines indicate fits of the form $r_g = A \log(t - t_0) + B$, for constants A and B. **(d-f)** Null habitats. The same as a-c but for randomized control habitats, constructed for each user by randomly reshuffling locations between habitats while preserving the number of habitats and the number of locations within each habitat. We see the pure logarithmic scaling of r_g is lost.

S6 Controls and hypothesis tests

We introduce Habitat controls to determine how meaningful the discovered habitats are, where we use different groupings of mobility locations to form control or null habitats. To compute habitat controls we form randomized habitats by shuffling locations between the original habitats at random in such a way as to preserve for each user both the number of habitats and the number of locations per habitat, strictly controlling for the size distributions of the habitats. These control for the nature of the groupings we find and whether or not it is meaningful for particular locations to share a habitat.

To begin, in Fig. Ia-c we show the temporal evolution of the gyradius $r_g(t)$ vs. t for the first three habitats. We study three sets of users, with total $R_g \approx 10$, 30, and 100 km, respectively. For all groups we see that $r_g \sim \log(t - t_0)$, amplifying the results from main text Fig. 3d. Meanwhile, in Fig. Id-f we show the same quantities but for the shuffled habitats where locations are randomized. We see that the pure logarithmic time evolution is lost, indicating that the evolution we observe is not due to the relative sizes (numbers of locations) of the habitats, nor to simply the number of habitats, but due more fundamentally to their spatial structure and the spatiotemporal flows of the users.

Meanwhile, the results from main text Fig. 5 show an intriguing relationship between human mobility and communication activity. Here we quantify the relationship using the Kendall's τ (tau-b) rank correlation coefficient [13], which is a nonparametric hypothesis test used to measure the association between two measured quantities. The coefficient $\tau > 0$ indicates positive association, $\tau < 0$ indicates negative association, and $\tau = 0$ indicates the absence of association.

Mobility is evaluated by the number of habitats N_H and the total gyradius R_g, while interaction concentration is quantified by the probability of calling the most frequent contact P_{MFC}, the cumulative probability of calling the top-three most frequently contacted partners C_{MFC}, and the total number of partners k.

Table A: Nonparametric correlations between mobility and communication. We quantify the relationship between mobility (columns) and communication (rows) using Kendall's τ rank correlation coefficient. User mobility is evaluated by the number of habitats N_{H} and the gyradius R_{g}, and their concentration is evaluated by P_{MFC}, C_{MFC} (the cumulative probability of calling any of the three most frequently contacted partners) and k (the number of partners). Each table entry shows the coefficient τ and its corresponding p-value (parenthesis). We see a negative association between N_{H} and P_{MFC} (and C_{MFC}), while N_{H} and k are positively correlated. An association between R_{g} and the communication measures appears absent. However, when separating users with a single habitat ($N_{\mathrm{H}} = 1$) from the rest ($N_{\mathrm{H}} > 1$), we see that the growth of R_{g} has two opposite trends: within a single habitat, R_{g} grows with P_{MFC}; when users have more than one habitat, P_{MFC} begin to drop. This implies a coupling between mobility habitats and interaction concentration, one that cannot be captured by R_{g} alone.

	Mobility			
Communication	N_{H}	R_{g}	R_{g} ($N_{\mathrm{H}} = 1$)	R_{g} ($N_{\mathrm{H}} > 1$)
P_{MFC}	-0.133 ($< 2.2 \times 10^{-16}$)	-0.00648 (0.0039)	0.354 ($< 2.2 \times 10^{-16}$)	-0.0324 ($< 2.2 \times 10^{-16}$)
C_{MFC}	-0.153 ($< 2.2 \times 10^{-16}$)	-0.0127 (1.7×10^{-8})	0.358 ($< 2.2 \times 10^{-16}$)	-0.0369 ($< 2.2 \times 10^{-16}$)
k	0.214 ($< 2.2 \times 10^{-16}$)	-0.0728 ($< 2.2 \times 10^{-16}$)	-0.44 ($< 2.2 \times 10^{-16}$)	-0.0619 ($< 2.2 \times 10^{-16}$)
$f_{\mathrm{reciprocal}}$	-0.0452 ($< 2.2 \times 10^{-16}$)	0.034 ($< 2.2 \times 10^{-16}$)	0.369 ($< 2.2 \times 10^{-16}$)	0.00462 (0.050929)

In Table A, we see a negative association between N_{H} and P_{MFC} (as well as C_{MFC}), while N_{H} and k are positively correlated. This suggests that people who are more habitually mobile (with more habitats) tend to distribute their communication over more contacted ties.

An association between R_{g} and the communication measures at first appears absent. However, when separating users who possess only a single habitat ($N_{\mathrm{H}} = 1$) from those who don't ($N_{\mathrm{H}} > 1$), we discover that the relationship with R_{g} shows two opposing trends: for users with a single habitat, R_{g} grows with P_{MFC}; when users have more than one habitat, their P_{MFC} begin to drop. These correlations suggest a coupling between mobility habitats and interaction concentration, which cannot be captured by a single R_{g} value. Similarly, for $f_{\mathrm{reciprocal}}$ the association with R_{g} is stronger when $N_{\mathrm{H}} = 1$, than when $N_{\mathrm{H}} > 1$.

References

[1] Onnela JP, Saramäki J, Hyvönen J, Szabó G, Lazer D, et al. (2007) Structure and tie strengths in mobile communication networks. Proceedings of the National Academy of Sciences 104: 7332.

[2] González MC, Hidalgo CA, Barabási AL (2008) Understanding individual human mobility patterns. Nature 453: 779–782.

[3] Song C, Qu Z, Blumm N, Barabási AL (2010) Limits of predictability in human mobility. Science 327: 1018.

[4] Song C, Koren T, Wang P, Barabási AL (2010) Modelling the scaling properties of human mobility. Nature Physics .

[5] Bagrow JP, Koren T (2009) Investigating bimodal clustering in human mobility. In: International Conference on Computational Science and Engineering, 2009. CSE'09. IEEE, volume 4, pp. 944–947.

[6] Bagrow JP, Wang D, Barabási AL (2011) Collective response of human populations to large-scale emergencies. PLoS ONE 6: e17680.

[7] Wasserman S, Faust K (1994) Social Network Analysis: Methods and Applications. Cambridge University Press, 1st edition.

[8] Di Battista G, Eades P, Tamassia R, Tollis IG (1999) Graph drawing: algorithms for the visualization of graphs, volume 3. Prentice Hall New Jersey, USA.

[9] Rosvall M, Bergstrom CT (2008) Maps of random walks on complex networks reveal community structure. Proceedings of the National Academy of Sciences 105: 1118.

[10] Fortunato S (2010) Community detection in graphs. Physics Reports 486: 75–174.

[11] Park J, Lee DS, González MC (2010) The eigenmode analysis of human motion. Journal of Statistical Mechanics: Theory and Experiment 2010: P11021.

[12] Dunbar R (1992) Neocortex size as a constraint on group size in primates. Journal of Human Evolution 22: 469–493.

[13] Kendall M, Gibbons JD (1990) Rank Correlation Methods. C. Griffin, 5th edition.
